# Pertuzumab, trastuzumab, and docetaxel for Chinese patients with previously untreated HER2-positive locally recurrent or metastatic breast cancer (PUFFIN): final analysis of a phase III, randomized, double-blind, placebo-controlled study

**DOI:** 10.1007/s10549-022-06775-1

**Published:** 2022-12-04

**Authors:** Binghe Xu, Wei Li, Qingyuan Zhang, Qiao Li, Xiaojia Wang, Huiping Li, Tao Sun, Yongmei Yin, Hong Zheng, Jifeng Feng, Huaqi Zhu, Asna Siddiqui, Harrison Macharia, Adam Knott

**Affiliations:** 1 Department of Medical Oncology National Cancer Center/Cancer Hospital, Chinese Academy of Medical Sciences, and Peking Union Medical College, Beijing, China; 2 The Cancer Center, The First Hospital of Jilin University, Changchun, Jilin China; 3 Harbin Medical University, Harbin, China; 4 National Cancer Center/National Clinical Research Center for Cancer/Cancer Hospital, Chinese Academy of Medical Sciences, and Peking Union Medical College, Beijing, China; 5 Zhejiang Cancer Hospital, Hangzhou City, China; 6 Key Laboratory of Carcinogenesis and Translational Research (Ministry of Education/Beijing), Department of Breast Oncology, Peking University Cancer Hospital and Institute, Beijing, China; 7 Breast Medicine, Cancer Hospital of China Medical University, Liaoning Cancer Hospital and Institute, Liaoning, China; 8 Jiangsu Province Hospital, Nanjing, China; 9 West China Hospital, Sichuan University, Chengdu, China; 10 Jiangsu Cancer Hospital, Nanjing, China; 11 Roche Pharma Product Development China, Shanghai, China; 12 Product Development Clinical Oncology, Roche Products Limited, Welwyn Garden City, UK; 13 Oncology Biostatistics, F. Hoffmann-La Roche Ltd, Basel, Switzerland; 14 Product Development Oncology, Roche Products Limited, Welwyn Garden City, UK

**Keywords:** Pertuzumab, Metastatic breast cancer, Locally recurrent breast cancer, HER2, Chinese, Clinical trial, Metastasis

## Abstract

**Purpose:**

PUFFIN (NCT02896855), a Chinese bridging study in patients with previously untreated HER2-positive locally recurrent or metastatic breast cancer, assessed consistency of efficacy and safety of pertuzumab plus trastuzumab and docetaxel versus placebo, trastuzumab, and docetaxel, with CLEOPATRA (NCT00567190).

**Methods:**

Eligible patients, *n* = 243, were randomized 1:1, stratified by visceral disease and hormone receptor status, to pertuzumab, trastuzumab, and docetaxel or placebo, trastuzumab, and docetaxel. Primary endpoint: investigator-assessed progression-free survival (PFS). Secondary endpoints: safety and overall survival (OS). After primary analysis, patients could cross over to the pertuzumab arm.

**Results:**

Updated median PFS: 16.5 months (pertuzumab arm) and 12.5 months (placebo arm), with a hazard ratio (HR) of 0.60 [95% confidence interval (CI) 0.45, 0.81; *p* = 0.0008]. Median OS was not reached in either arm; the OS HR was 0.68 (95% CI 0.45, 1.03; *p* = 0.0658). Safety was similar in both arms with no new safety signals: 73.8% (pertuzumab arm) and 69.2% (placebo arm) experienced grade ≥ 3 adverse events. No heart failure, symptomatic left ventricular systolic dysfunction, or left ventricular ejection fraction decline of < 40% were reported.

**Conclusions:**

The PUFFIN final analysis showed, per the primary analysis, that overall efficacy of pertuzumab plus trastuzumab and docetaxel was consistent with CLEOPATRA. Safety remained consistent with the known pertuzumab profile. Overall, PUFFIN contributes to the totality of data with pertuzumab in previously untreated HER2-positive locally recurrent or metastatic breast cancer and supports the favorable benefit–risk profile of pertuzumab in Chinese patients.

*Trial registration*: ClinicalTrials.gov, NCT02896855, registered 7 September 2016.

## Introduction

Pertuzumab and trastuzumab (PERJETA^®^ and Herceptin^®^; F. Hoffmann-La Roche Ltd, Basel, Switzerland) are monoclonal antibodies that bind to different domains on human epidermal growth factor receptor 2 (HER2). The difference in binding creates complementary antitumor activity by inhibiting signaling and promoting antibody-dependent cellular cytotoxicity [1, 2].

Pertuzumab plus trastuzumab and docetaxel in patients with previously untreated HER2-positive locally recurrent or metastatic breast cancer was shown to improve progression-free and overall survival (PFS and OS) significantly compared with placebo plus trastuzumab and docetaxel in CLEOPATRA (NCT00567190). At CLEOPATRA’s primary analysis, independently assessed median PFS was 18.5 months in the pertuzumab arm and 12.4 months in the placebo arm. The PFS hazard ratio (HR) was 0.62 [95% confidence interval (CI) 0.51, 0.75; *p* < 0.001] and OS data were immature at the first analysis [3]. At the second OS analysis, median OS was immature in the pertuzumab arm and 37.6 months in the placebo arm, with a HR of 0.66 (95% CI 0.52, 0.84; *p* = 0.0008) [4]. In the final OS analysis, median OS was 56.5 months and 40.8 months in the respective arms, with a HR of 0.68 (95% CI 0.56, 0.84; *p* < 0.001) [5]. At the 8-year end-of-study analysis, median OS was 57.1 months and 40.8 months, respectively, with a HR of 0.69 (95% CI 0.58, 0.82; *p* < 0.0001) [6].

Exploratory analyses of safety showed a higher proportion of patients from Asia [China (including Hong Kong) plus Japan, Korea, the Philippines, Singapore, and Thailand] experienced adverse events (AEs) than patients from Europe, North America, and South America combined. The most common AE was febrile neutropenia, which the authors postulated may have been related to increased diarrhea and mucosal inflammation. Despite these differences, comparable survival benefits across regions were observed [7].

PUFFIN (NCT02896855) was a Chinese bridging study to evaluate the efficacy and safety of pertuzumab and to determine consistency with CLEOPATRA. The primary analysis showed that efficacy data were consistent with CLEOPATRA. Median investigator-assessed PFS was 14.5 months and 12.4 months in the pertuzumab and placebo arms, respectively; OS was relatively immature. The PFS HR was 0.69 (95% CI 0.49, 0.99), which was similar to the investigator-assessed PFS HR in CLEOPATRA; 0.62 (95% CI 0.51, 0.75) [3]. Safety was consistent with the known pertuzumab profile [8]. We present the final analysis of PUFFIN, including updated PFS, OS, and safety data, with an additional 20 + months of follow-up of efficacy and AE reporting.

## Methods

PUFFIN was a phase III, randomized, double-blind, placebo-controlled study conducted across 15 centers in China. The protocol was approved by the institutional review board at each participating site. Informed consent was provided from all participants. The study design, eligibility criteria, procedures, assessments, and statistical methods have been reported previously in the primary analysis [8].

Eligible patients were aged ≥ 18 years with HER2-positive (centrally confirmed immunohistochemistry 3 + or in situ hybridization-positive), locally recurrent or metastatic breast cancer. Patients were eligible if they had not received prior treatment for metastatic disease (except for one hormonal regimen before randomization), no prior treatment with HER2-targeting therapies (except trastuzumab in the neoadjuvant or adjuvant settings) or tyrosine kinase inhibitors, and were disease-free for ≥ 12 months. Hormone receptor status was centrally confirmed. Further inclusion criteria included a left ventricular ejection fraction (LVEF) of ≥ 55% at baseline and an Eastern Cooperative Oncology Group Performance Status (ECOG PS) of 0 or 1. Patients were eligible if they had measurable or non-measurable disease.

Patients were excluded if they had prior exposure to doxorubicin of ≥ 360 mg/m^2^, had a history of LVEF decline to < 50% during or after trastuzumab in the neoadjuvant or adjuvant settings, or if they had any other conditions that were not controlled and could affect the patient’s ability to comply with the study.

### Procedures

Randomization was 1:1, using visceral disease and hormone receptor status to stratify patients to pertuzumab (840 mg loading dose, followed by 420 mg every 3 weeks), trastuzumab (8 mg/kg loading dose, then 6 mg/kg every 3 weeks), and docetaxel (75 mg/m^2^ every 3 weeks) or placebo, trastuzumab, and docetaxel arms; all drugs were given intravenously. The HER2-targeted therapies were given until disease progression or unacceptable toxicity. The docetaxel dose could be reduced to 55 mg/m^2^ if febrile neutropenia or severe or cumulative cutaneous reactions occurred. Discontinuation of docetaxel was at the discretion of the patient and treating physician after completion of cycle 6.

After the primary analysis, patients could cross over from the placebo arm to the pertuzumab arm due to pertuzumab showing a clinically significant improvement over placebo.

### Statistical methods

PFS and OS were estimated using the Kaplan–Meier approach. HR and 95% CIs were estimated by a Cox proportional hazard model using the stratification factors. To compare the two arms, a two-sided stratified log-rank test was used. Statistical testing was considered exploratory. Safety analyses were descriptive.

## Results

### Study population

**Fig. 1:**
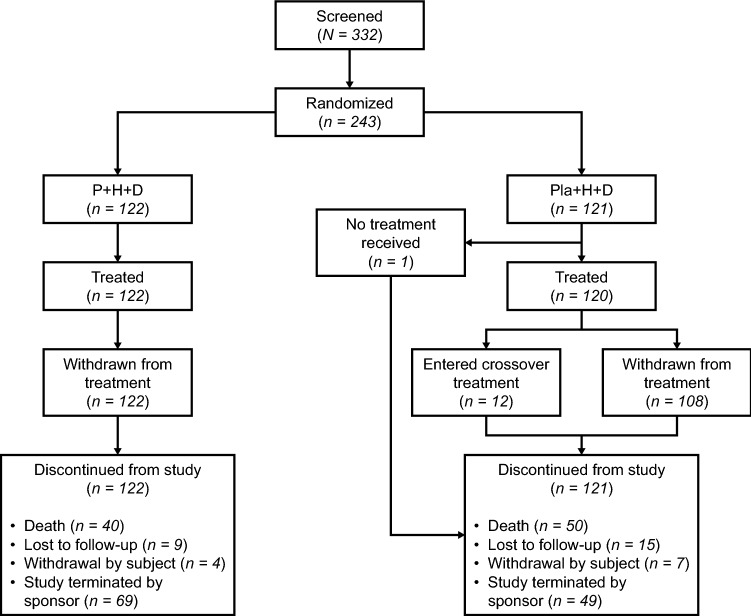
Disposition of patients

From 13 September 2016 to 28 September 2017, 243 eligible patients were randomized: 122 and 121 to the pertuzumab and placebo arms, respectively [8], as shown in Fig. 1. One patient randomized to the placebo arm discontinued prior to treatment. The safety population comprised 122 and 120 patients in the respective arms. After the primary analysis, 12 patients crossed over from the placebo arm to the pertuzumab arm. Clinical cut-off for this final analysis was 23 October 2020. Median follow-up was 39.3 months in the pertuzumab arm and 33.4 months in the placebo arm. Baseline demographics and disease characteristics were balanced between arms [8]. Eleven patients received ovarian function suppressors, two of whom received them during the study. Five patients underwent an oophorectomy, only one during the study.

### Progression-free survival

**Fig. 2:**
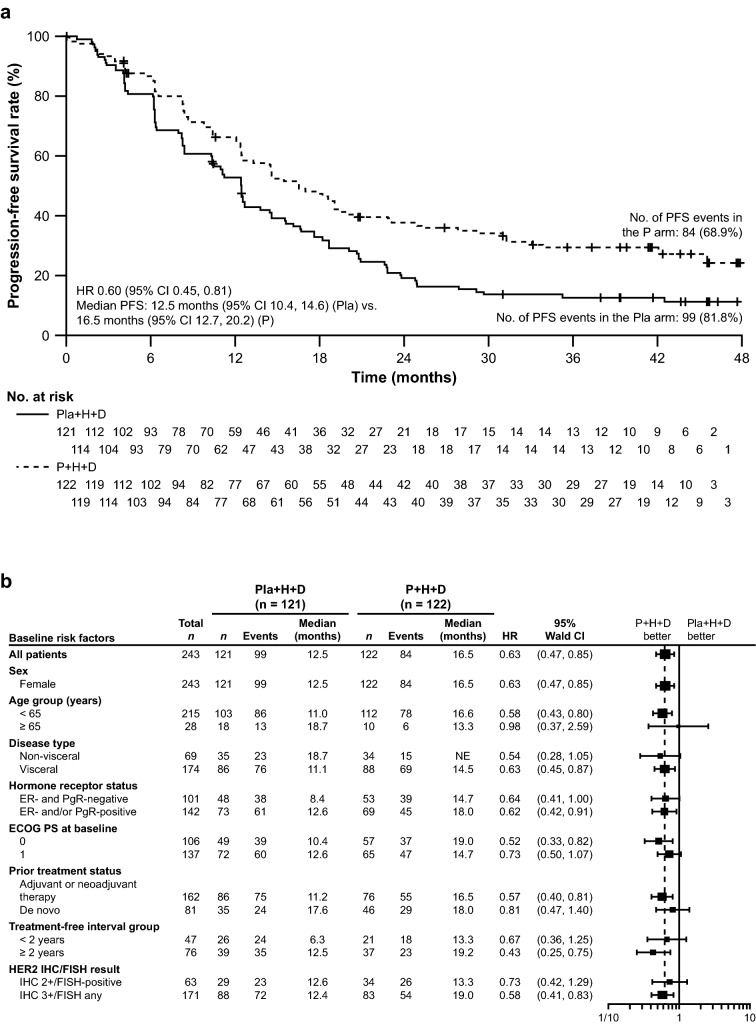
Investigator-assessed PFS in **a** the intention-to-treat population and **b** subgroups. *CI* confidence interval, *D* docetaxel, *ECOG PS* Eastern Cooperative Oncology Group Performance Status, *ER* estrogen receptor, *FISH* fluorescence in situ hybridization, *H* trastuzumab, *HER2* human epidermal growth factor receptor 2, *HR* hazard ratio, *IHC* immunohistochemistry, *P* pertuzumab, *PFS*, progression-free survival, *PgR* progesterone receptor, *Pla* placebo

Updated median investigator-assessed PFS was 16.5 months (95% CI 12.7, 20.2) in the pertuzumab arm and 12.5 months (95% CI 10.4, 14.6) in the placebo arm (Fig. 2a). The stratified HR was 0.60 (95% CI 0.45, 0.81; *p* = 0.008) and the unstratified HR was 0.63 (95% CI 0.47, 0.85; *p* = 0.0019). The number of patients who had a PFS event was 84/122 (68.9%) in the pertuzumab arm and 99/120 (81.8%) in the placebo arm. Subgroup analyses are shown in Fig. 2b and were consistent with the overall results.

### Overall survival

**Fig. 3:**
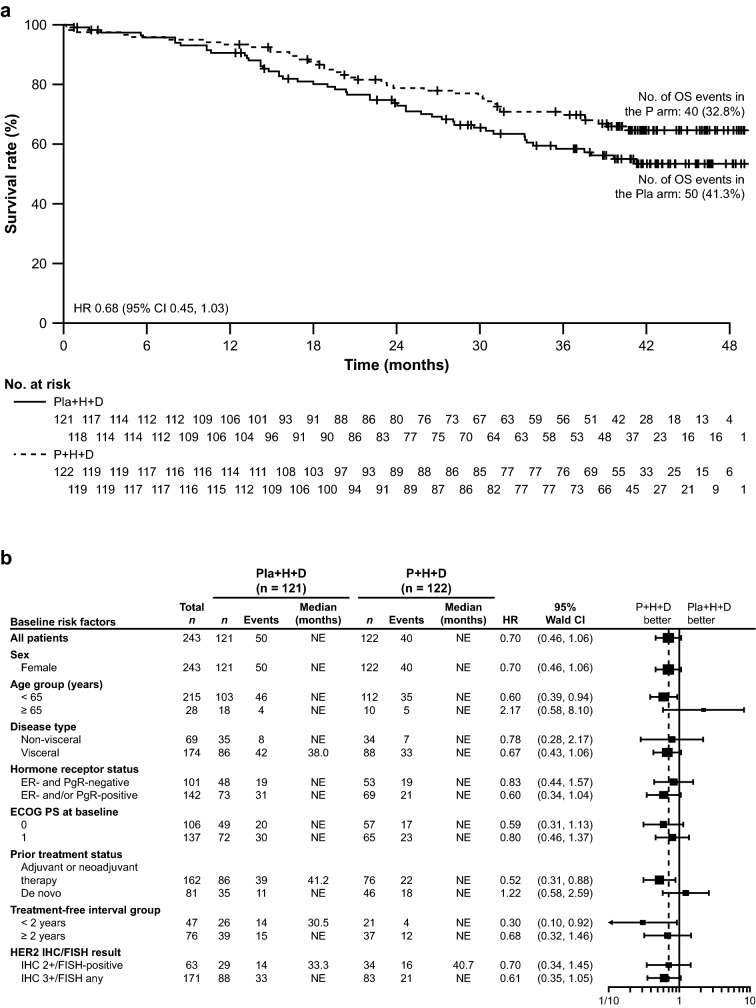
OS in **a** the intention-to-treat population and **b** subgroups. *CI* confidence interval, *D* docetaxel, *ECOG PS* Eastern Cooperative Oncology Group Performance Status, *ER* estrogen receptor, *FISH* fluorescence in situ hybridization, *H* trastuzumab, *HER2* human epidermal growth factor receptor 2, *HR* hazard ratio, *IHC* immunohistochemistry, *OS* overall survival, *P* pertuzumab, *PgR* progesterone receptor, *Pla* placebo

Median OS was not reached in either arm. The stratified HR was 0.68 (95% CI 0.45, 1.03; *p* = 0.0658) and the unstratified HR was 0.70 (95% CI 0.46, 1.06; *p* = 0.0864) (Fig. 3a). There were 40 OS events (32.8%) in the pertuzumab arm and 50 (41.3%) in the placebo arm. Subgroup analyses are shown in Fig. 3b and were consistent with the overall results.

### Treatment exposure

Prior to crossover, the median number of pertuzumab or placebo cycles administered to patients was 22.0 (range 1–4) and 17.0 (range 1–68), over the respective median duration of 66.1 weeks (range 3–225) and 52.3 weeks (range 3–207). The median number of trastuzumab cycles in the pertuzumab and placebo arms was 22.0 (range 1–74) and 17.0 (range 1–74), respectively. Both arms had a similar median number of docetaxel cycles, with 7.0 (range 1–26) in the pertuzumab arm and 6.5 (range 1–22) in the placebo arm. The median dose of each was 900.0 mg and 826.1 mg. Following crossover, the median number of pertuzumab cycles was 6.0 (range 4–8), with a median duration of 18.1 weeks (range 12–24).

### Safety

A summary of the safety profile is shown in Table 1. The number of patients who experienced ≥ 1 AE was 121/122 (99.2%) in the pertuzumab arm and 115/120 (95.8%) in the placebo arm. The most common AE in both arms was leukopenia, reported in 91/122 patients (74.6%) and 87/120 patients (72.5%), respectively. In the pertuzumab arm, AEs with an incidence ≥ 5% higher than with placebo were anemia, alopecia, diarrhea, pyrexia, cough, hypokalemia, hyperglycemia, and stomatitis. There were 111/122 patients (91.0%) in the pertuzumab arm and 104/120 patients (86.7%) in the placebo arm who experienced AEs with a causal relationship to HER2-targeted therapies. Grade ≥ 3 AEs were experienced by 90 patients (73.8%) in the pertuzumab arm and 83 patients (69.2%) in the placebo arm, with the most common being blood and lymphatic system disorders, particularly neutropenia and leukopenia. The number of patients who experienced serious AEs was 30 (24.6%) in the pertuzumab arm and 23 (19.2%) in the placebo arm; the most common were neutropenia and febrile neutropenia. The number of patients who withdrew from treatment due to AEs relating to either pertuzumab or placebo was seven (5.7%) and two (1.7%), respectively. AEs were associated with the deaths of two patients (1.7%) in the placebo arm and four patients (3.3%) in the pertuzumab arm. AEs of interest to pertuzumab are reported in Table 2. No heart failure, symptomatic left ventricle systolic dysfunction, or LVEF decline of < 40% were reported; two patients in the pertuzumab arm experienced grade 2 ejection fraction decreases, which were reported as AEs of special interest, and led to the discontinuation of pertuzumab in both patients. A safety summary for the crossover population is shown in Table 3. There were very few AEs in the crossover group, with five (41.7%) patients experiencing ≥ 1 AE, with only one grade ≥ 3 AE (large intestine polyp).

**Table 1:** Safety summary in the safety population prior to crossover

	Pertuzumab plus trastuzumab plus docetaxel (*n* = 122)	Placebo plus trastuzumab plus docetaxel (*n* = 120)
Grade ≥ 1 AEs	121 (99.2)	115 (95.8)
AEs leading to death	2 (1.7)	4 (3.3)
Grade ≥ 3 AEs	90 (73.8)	83 (69.2)
AEs leading to withdrawal from pertuzumab or placebo	7 (5.7)	2 (1.7)
Serious AEs	30 (24.6)	23 (19.2)
Most common AEs (all grades)^a^		
Leukopenia	91 (74.6)	87 (72.5)
Neutropenia	88 (72.1)	85 (70.8)
Anemia	67 (54.9)	58 (48.3)
Alanine aminotransferase increased	45 (36.9)	56 (46.7)
Aspartate aminotransferase increased	45 (36.9)	49 (40.8)
Alopecia	50 (41.0)	40 (33.3)
Diarrhea	56 (45.9)	28 (23.3)
Pyrexia	29 (23.8)	21 (17.5)
Asthenia	25 (20.5)	20 (16.7)
Cough	26 (21.3)	17 (14.2)
Pain	19 (15.6)	22 (18.3)
Upper respiratory tract infection	18 (14.8)	16 (13.3)
Nausea	15 (12.3)	15 (12.5)
Edema peripheral	12 (9.8)	18 (15.0)
Decreased appetite	15 (12.3)	14 (11.7)
Blood bilirubin increased	14 (11.5)	14 (11.7)
Nail discoloration	12 (9.8)	15 (12.5)
Hypokalemia	18 (14.8)	8 (6.7)
Vomiting	13 (10.7)	11 (9.2)
Hyperglycemia	14 (11.5)	8 (6.7)
Hypoesthesia	13 (10.7)	8 (6.7)
Thrombocytopenia	9 (7.4)	12 (10.0)
Weight increased	4 (3.3)	16 (13.3)
Stomatitis	15 (12.3)	4 (3.3)
Most common grade ≥ 3 AEs^b^		
Neutropenia	71 (58.2)	70 (58.3)
Leukopenia	60 (49.2)	57 (47.5)
Febrile neutropenia	5 (4.1)	5 (4.2)
Hypertension	5 (4.1)	3 (2.5)
Anemia	6 (4.9)	1 (0.8)
Diarrhea	5 (4.1)	2 (1.7)
Pneumonia	4 (3.3)	1 (0.8)

Data are number of patients, *n* (%)

Table includes AEs with onset from first dose of study drug through 42 days after last dose of study drug

*AE* adverse event

^a^Reported in ≥ 10% of patients in either arm

^b^Reported in ≥ 3% of patients in either arm

**Table 2:** AEs of interest to pertuzumab therapy in the safety population

	Pertuzumab plus trastuzumab plus docetaxel (*n* = 122)	Placebo plus trastuzumab plus docetaxel (*n* = 120)
Diarrhea	56 (45.9)	28 (23.3)
Grade ≥ 3	5 (4.1)	2 (1.7)
Rash	36 (29.5)	22 (18.3)
Grade ≥ 3	2 (1.6)	3 (2.5)
Leukopenia	96 (78.7)	89 (74.2)
Grade ≥ 3	74 (60.7)	73 (60.8)
Leukopenia infection	6 (4.9)	3 (2.5)
Grade ≥ 3	1 (0.8)	0
Febrile neutropenia	5 (4.1)	5 (4.2)
Grade ≥ 3	5 (4.1)	5 (4.2)
Febrile neutropenia infection	0	0
Grade ≥ 3	0	0
Anaphylaxis and hypersensitivity	2 (1.6)	1 (0.8)
Grade ≥ 3	0	1 (0.8)
Infusion-related reactions	41 (33.6)	29 (24.2)
Grade ≥ 3	4 (3.3)	3 (2.5)
Mucositis	32 (26.2)	12 (10.0)
Grade ≥ 3	4 (3.3)	0
Interstitial lung disease	2 (1.6)	4 (3.3)
Grade ≥ 3	0	0

Data are number of patients, *n* (%)

AEs to monitor, i.e., AEs that the health authorities requested to be monitored closely (usually potential risks or missing information)

*AE* adverse event

**Table 3:** Safety summary in the safety population who crossed over to pertuzumab

	Pertuzumab plus trastuzumab plus docetaxel (*n* = 12)
Patients with ≥ 1 AEs	5 (41.7)
AEs (all grades)	
Aspartate aminotransferase increase	2 (16.7)
Alanine aminotransferase increase	1 (8.3)
Blood alkaline phosphate increase	1 (8.3)
Blood creatinine increase	1 (8.3)
Hyperglycemia	1 (8.3)
Hypoalbuminemia	1 (8.3)
Hypokalemia	1 (8.3)
Large intestine polyp	1 (8.3)
Neutropenia	1 (8.3)
Peripheral arterial occlusion disease	1 (8.3)
Grade ≥ 3 AEs	
Large intestine polyp	1 (8.3)

Data are number of patients, *n* (%)

Table includes AEs with onset from first dose of study drug through 42 days after last dose of study drug

*AE* adverse event

## Discussion

PUFFIN previously met its primary objectives, demonstrating consistency of efficacy with CLEOPATRA [8]. The stratified PFS HR had decreased since the primary analysis [0.60 (95% CI 0.45, 0.81) and 0.69 (95% CI 0.49, 0.99), respectively], with an increased reliability of a narrower CI, and remained consistent with the CLEOPATRA intention-to-treat (ITT) population [HR = 0.62 (95% CI 0.51, 0.75)] [3, 8]. The updated median PFS was closer to the CLEOPATRA ITT population, further demonstrating consistency. As discussed in the primary manuscript, it is important to note that PFS was investigator-assessed per Response Evaluation Criteria in Solid Tumors (RECIST) v1.1 in PUFFIN, whereas in CLEOPATRA, PFS was independent review facility-assessed per RECIST v1.0. The OS HR values were also similar between PUFFIN and CLEOPATRA’s second OS analysis [4]. However, CLEOPATRA had a longer follow-up period; therefore, PUFFIN would require a longer follow-up period to show greater differences between arms and to evaluate median OS. The OS analyses were based on the ITT population, the 12 crossover patients were included in the placebo group for the analysis as randomly assigned. Given the small number of crossover patients, these analyses were not adjusted for crossover to the pertuzumab group. However, they are likely to be conservative in estimating the overall treatment effect.

Subgroup analyses showed consistency with the overall PFS and OS results. Compared with the primary PFS subgroup analysis, the final analysis showed an overall shift toward the pertuzumab group having improved PFS and OS, with little difference between disease type, hormone receptor status, and low or high HER2 status. Patients aged ≥ 65 years showed no difference in PFS between arms. Subgroup analyses showed that patients in the < 65 years age group [PFS HR = 0.58 (95% CI 0.43, 0.80) and OS HR = 0.60 (95% CI 0.39, 0.94)] had better outcomes with pertuzumab. Patients who were ≥ 2 years treatment-free had improved PFS over patients who were < 2 years treatment-free [HR = 0.43 (95% CI 0.25, 0.75) vs. HR = 0.67 (95% CI 0.36, 1.25)]. However, the OS subgroup analysis showed patients who were < 2 years treatment-free had improved OS over patients who were ≥ 2 years treatment-free [HR = 0.30 (95% CI 0.10, 0.92) vs. HR = 0.68 (95% CI 0.32, 1.46)]. Patients with de novo disease had poorer OS with pertuzumab compared with placebo [HR = 1.22 (95% CI 0.58, 2.59)]; this conflicts with CLEOPATRA [HR = 0.65 (95% CI 0.51, 0.82)], which showed that patients with no previous treatment had better prognosis with pertuzumab than with placebo [6]. Additionally, both CLEOPATRA and a retrospective study of patients with de novo, HER2-positive, metastatic breast cancer concluded that trastuzumab as a first-line treatment can improve OS in these patients [6, 9]. Despite PUFFIN showing poorer OS in a subset of patients, new anti-HER2 therapies and regimens may provide improved clinical outcomes following disease progression [10–13]. Furthermore, the development of screening techniques (such as liquid biopsy) can identify targetable genomic alterations that can be utilized to determine an optimal treatment regimen in patients [14].

The overall safety profile was consistent with the primary analysis, with few AEs reported that required additional follow-up in either arm, and no new safety signals. Leukopenia remained the most common AE in the study, followed by neutropenia. Grade ≥ 3 AEs remained balanced between arms, with neutropenia, leukopenia, febrile neutropenia, anemia, and diarrhea being the most reported (with the addition of hypertension and pneumonia since the primary analysis). No new cardiac AEs were reported since the primary analysis. Comparing the safety profile to the Asian population in CLEOPATRA’s exploratory analysis, the rates of AEs of special interest to pertuzumab were lower, specifically diarrhea (45.9% vs. 74.4% vs.) and febrile neutropenia (4.1% vs. 25.6%) [7]. This suggests that AE management has improved over time, with rates similar to the overall population in CLEOPATRA (diarrhea 66.8% and febrile neutropenia 13.8%) [3]. Therefore, the safety profile remained consistent with that observed in the primary analysis and CLEOPATRA ITT population, and with the established pertuzumab safety profile.

## Conclusions

This final analysis of PUFFIN showed, as with the primary analysis, that overall efficacy was consistent with that of the ITT population in CLEOPATRA. Safety of pertuzumab remained consistent with the known pertuzumab profile. Overall, PUFFIN contributes to the totality of data with pertuzumab in previously untreated HER2-positive locally recurrent or metastatic breast cancer and supports the favorable benefit–risk profile of pertuzumab in Chinese patients.

## Data Availability

For eligible studies, qualified researchers may request access to individual patient-level clinical data through a data request platform. At the time of writing, this request platform is Vivli. https://vivli.org/ourmember/roche/. For up-to-date details on Roche’s Global Policy on the Sharing of Clinical Information and how to request access to related clinical study documents, see here: https://go.roche.com/data_sharing. Anonymized records for individual patients across more than one data source external to Roche cannot, and should not, be linked due to a potential increase in risk of patient re-identification.

## Abbreviations

AE: Adverse event
CI: Confidence interval
D: Docetaxel
ECOG PS: Eastern Cooperative Oncology Group Performance Status
ER: Estrogen receptor
FISH: Fluorescence in situ hybridization
H: Trastuzumab
HER2: Human epidermal growth factor receptor 2
HR: Hazard ratio
ICH: International Council for Harmonisation
IEC: Independent ethics committee
IHC: Immunohistochemistry
ITT: Intention-to-treat
LVEF: Left ventricular ejection fraction
OS: Overall survival
P: Pertuzumab
PFS: Progression-free survival
PgR: Progesterone receptor
Pla: Placebo
RECIST: Response Evaluation Criteria in Solid Tumors
